# Effects of Changed Aircraft Noise Exposure on the Use of Outdoor Recreational Areas

**DOI:** 10.3390/ijerph7113890

**Published:** 2010-11-03

**Authors:** Norun Hjertager Krog, Bo Engdahl, Kristian Tambs

**Affiliations:** 1 Department of Air Pollution and Noise, Division of Environmental Medicine, Norwegian Institute of Public Health, PO Box 4404 Nydalen, N-0403 Oslo, Norway; 2 Division of Mental Health, Norwegian Institute of Public Health, PO Box 4404 Nydalen, N-0403 Oslo, Norway; E-Mails: bo.engdahl@fhi.no (B.E.); kristian.tambs@fhi.no (K.T.)

**Keywords:** aircraft noise, annoyance, behavioural effects, constraints, displacement, panel study, place attachment, outdoor recreation, telephone survey

## Abstract

This paper examines behavioural responses to changes in aircraft noise exposure in local outdoor recreational areas near airports. Results from a panel study conducted in conjunction with the relocation of Norway’s main airport in 1998 are presented. One recreational area was studied at each airport site. The samples (*n* = 1,264/1,370) were telephone interviewed about their use of the area before and after the change. Results indicate that changed aircraft noise exposure may influence individual choices to use local outdoor recreational areas, suggesting that careful considerations are needed in the planning of air routes over local outdoor recreational areas. However, considerable stability in use, and also fluctuations in use unrelated to the changes in noise conditions were found. Future studies of noise impacts should examine a broader set of coping mechanisms, like intra- and temporal displacement. Also, the role of place attachment, and the substitutability of local areas should be studied.

## 1. Introduction and Review of the Literature

The purpose of this paper is to examine the influence of noise from commercial air traffic on individual choices to visit specific local outdoor recreational areas. Although the experience of natural quiet and the absence of noise are indicated to be among the most important motivations for visiting outdoor recreational areas [1,2], studies on behavioural consequences of noise on outdoor recreation are largely lacking. In a qualitative explorative study of hypothetical site choices [3] peacefulness and seclusion were attributes that strongly attracted several subjects, while the presence of motorized vehicles was rated negatively because of the noise that follows. In evaluating the single aircraft overflight they remembered best, almost 10 percent of the visitors to four wilderness areas responded that “it made me (slightly to extremely) feel like changing my travel plans” [4]. Fidell *et al.* [5] found no significant relationships between intention to revisit and annoyance due to sight or sound of aircraft in two studies of effects of aircraft overflights on wilderness recreationists. But none of these cross sectional studies examined actual revisit behaviour.

Various kinds of adverse experiential effects of aircraft noise on outdoor recreationists, like annoyance or being bothered, have been demonstrated in field studies [5–14]. Other effects found are interference with natural quiet [6,8,14] or detraction from the recreational experience [9]. Studies of conflict between different user groups in outdoor recreational areas have often focused on motorized *versus* non-motorized use [15–20]. An asymmetrical relationship has been found where motorized use interferes with the goals of other recreationists. The word “noise” is not always mentioned in these studies, but may more or less be an implicit or underlying issue. However, examining reactions to different aspects of snowmobiles, both Jackson and Wong [19] and Vittersø *et al.* [20] found noise to be the most salient problem.

Factors that adversely influence the participation in and enjoyment of recreational activities have been studied within constraints research. Jackson [21] offers the following description of constraints research: “Leisure constraints research aims to investigate “factors that are assumed by researchers and/or perceived or experienced by individuals to limit the formation of leisure preferences and/or to inhibit or prohibit participation and enjoyment in leisure” (modified from Jackson, 1991, 1997)” [22,23]. McClaskie *et al.* [24] recommended that qualitative aspects of available recreation opportunities should be investigated as barriers to participation in outdoor recreational activities. “Facilities” was one of the six dimensions of constraints identified by Jackson [25]. However, the facility related items that have been measured are few and general. Commonly, the items included describing qualitative aspects of the recreational resources are “facilities or areas are overcrowded” and/or “facilities or areas are poorly kept or maintained” [25–36]. McGuire [26] included an item “The places to do the activity have pollution problems”, but we have not found any study within this area of leisure research that examined noise as a possible constraint to the use of specific outdoor recreational areas. Overall, the focus of the constraints literature seems to be more on the formation of leisure choices in general, or the participation in special activities, than on the choice of specific facilities (or recreational areas) for performing the activity.

A relationship between the experience of adverse environmental factors at specific recreational sites and behavioural responses has been indicated in studies of displacement. Various specifications have been offered, but most broadly, the concept “displacement” describes behavioural changes related to any perceived adverse change in the recreational environment [37]. Displacement may involve a temporal or a spatial shift in the use of a site. Further, the spatial shift may be of two kinds, described as intra-site displacement or inter-site displacement. Intra-site displacement is a shift within a recreational area, while inter-site displacement means a shift from one recreational area to another [38]. The typology was adopted and developed from recreation substitutability research [39,40]. Behavioural responses to adverse area conditions have mainly been studied in relation to growths in use level [41,42], together with other conditions related to the recreational use of the area [37,38,43–50]. Results from the studies that have examined other factors in addition to use level and crowding point to the importance of attending to a broader set of setting elements in studies of displacement [37,50,51]. Findings from an early study by Anderson and Brown [37] even suggested that crowding, operationalized as visual encounters with others, might not be the most important factor in relation to displacement. However, as the authors state, the other factors studied were perhaps not really separate factors, but could be conceptualized as different manifestations of crowding (litter, noisy people, over-use).

To the extent that noise has been included in other studies of coping with adverse area conditions, the noise source has mainly been related to the recreational activity in the area [37,38,45], and it has not been very well described or specified. Becker [43] only refers to “too much noise”, without any further explanation, in his examination of factors that might influence river use. Noise was not found to be of significant influence in this study. “Too much noise” (related to the recreational use of the area) and “no quiet place to fish” were among the reasons given for decreased use of a popular reservoir in Oregon [38], although these were not the most frequently mentioned reasons. “Human-caused noise” was one of the factors identified to detract from the recreational experience and thereby cause stress in a study of stress and coping in outdoor recreational settings [45]. In a study of river use, “use of motors” was indicated to be the second most common cause (next to “too many people”) to run the river less, and also the second most common cause to run another river instead [50]. Noise was not explicitly mentioned in Shelby’s study, but one might suspect that noise was at least part of the problem.

Because of the indicated importance to outdoor recreationists of escaping from noise and pollution [1,2], and the adverse experiential effects of mechanical noise that have been demonstrated in the literature (e.g., [5–7]), it is also important to conduct studies that examine especially the behavioural responses to environmental noise that is not related to the recreational use of the areas. Aircraft noise has the potential to interfere with the recreational experience even in remote areas. Most studies that have been conducted on the effects of aircraft noise on outdoor recreationists have examined the effects in National Parks, wildernesses, and mountain areas [5–9,12]. That is, the kinds of areas that have been studied are areas that people typically travel for a distance to visit. The quality of local outdoor recreational areas must be assumed to be of no less importance than the quality of the national and internationally important areas, however, since foremost local areas offer people the opportunity for outdoor recreation on a regular basis in everyday life. As stated by Manning and Valliere [46]: “Local residents may comprise an especially interesting population for a study of coping in outdoor recreation because they are likely to use their local park often for recreation and they are likely to have used the park over a relatively long period of time.” Opportunities for relief from noise while visiting outdoor recreational areas might be especially important to people living in noisy areas, like in the vicinity of airports.

The relocation of Norway’s main airport in 1998 offered a unique opportunity to examine behavioural responses to changed noise exposure in local outdoor recreational areas. Since both the old and the new airport were situated in the vicinity of locally important outdoor recreational areas, effects of both a decrease and an increase in noise exposure could be studied. The study presented in this paper examines behavioural consequences of changed aircraft noise exposure through a panel study that captures the individual choices of the (potential) visitors to local outdoor recreational areas. The same samples of residents from the vicinity of the two study areas were telephone interviewed before and after the relocation of the airport. The present study is part of a larger empirical work carried out in connection with the relocation of the main airport, [10,11,52].

### Objectives and Research Questions

The purpose of the present paper is to offer an explorative contextual analysis of the relationship between aircraft noise exposure and behavioural reactions. The relationship between changed noise exposure and behavioural responses is analyzed in relation to visitor characteristics as well as other possible reasons for non-use. More specifically, the following questions are analyzed:

#### Effects of Decreased Noise Exposure

Was the area visited more frequently following the decrease in aircraft noise exposure by recreationists who also used the area before the change? Is there a relationship between aircraft noise being a reason for not using the area before the change, and becoming a visitor after the change? In the group of new visitors after the change, is there a relationship between frequency of use, and having reported aircraft noise to be a reason for not visiting the area before the change?

#### Effects of Increased Noise Exposure

Was the area visited less frequently following the increase in aircraft noise exposure by recreationists who also used the area before the change? What characteristics of the visitors increased the probability of being annoyed by sound from aircraft before the change? Is there a relationship between the visitors’ characteristics, and the probability of becoming a non-visitor after the change? In the group of new non-visitors after the change, is there a relationship between visitor characteristics before the change, and reporting aircraft noise to be a reason for not visiting the area?

## 2. Method

### 2.1. Study Areas

**Figure 1:**
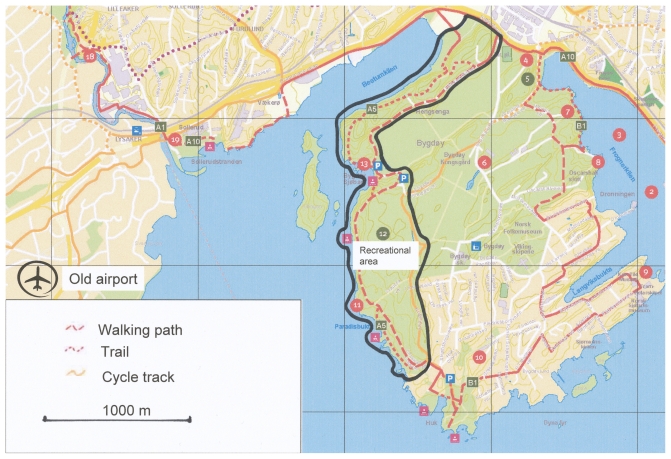
Map of the recreational area at the peninsula Bygdøy and its location relative to the old airport. Based on the Norwegian Mapping Authority—NE12000-271010SAS and walking map, Oslo West [55], published with permission from the Agency for Outdoor Recreation and Nature Management, City of Oslo.

**Figure 2:**
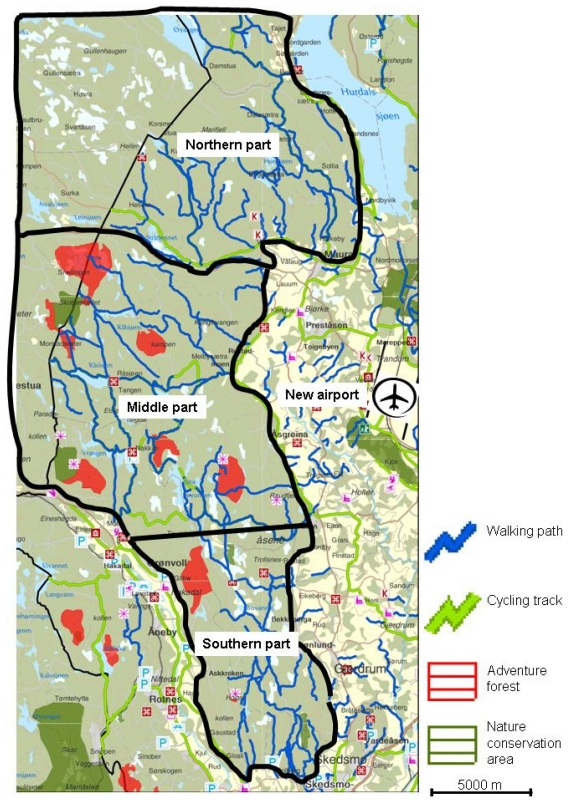
Map of the recreational area Romeriksåsen and its location relative to the new airport. Based on the Norwegian Mapping Authority—NE12000-271010SAS and walking map, Akershus [56], published with permission from Akershus County Council.

The two forest areas studied were Bygdøy, near the old main airport, and Romeriksåsen near the new airport. The forest area at Bygdøy is about 2.6 km^2^, while Romeriksåsen is about 7,600 km^2^. The areas were chosen on grounds of their location relative to the airports, and because they were much used by the local communities. Figures 1 and 2 show maps of the areas, and their location relative to the old and the new airport, respectively. Neither Bygdøy nor Romeriksåsen is situated more than about 11 kilometers from the homes of any of the respondents in the respective samples, which means that it is theoretically possible for them to use the area regularly. The air traffic over both areas was dominated by jet aircraft in route traffic both before and after the change. The change in exposure levels was largest at the old airport, since this airport was totally closed down. What remained after the change were overflights at high altitudes, and helicopter traffic. The new main airport was an existing airport, which was expanded. The areas and the aircraft noise levels are described at more detail elsewhere [10,11,53,54].

### 2.2. Procedure

Telephone interviews were conducted with the same people at the same time of the year before and after the moving of the airport, in November 1997 and 1999 for Romeriksåsen, and in May–June 1998 and 1999 for Bygdøy. Throughout the paper we will use the term t_1_ (time 1) whenever referring to data from the “before” situation in each of the areas, and t_2_ (time 2) whenever referring to the “after” situation. Because the data collection period each time lasted for about four weeks, people interviewed during the first half of the period the first time would also be interviewed during the first half of the period the second time, and *vice versa*. This was done to secure that changes in frequency of use were not due to seasonal changes.

The main part of the questionnaire was presented only to the visitors of the areas [visitors should have visited the area at least once during the past three months, and at least two times during the past six months (Romeriksåsen), or two times during the past 12 months (Bygdøy)]. Another set of questions was posed to a sub-sample of non-visitors. Visitors were interviewed about their use of the area the past three months, while non-visitors were asked why they had not visited the area during the same period. At the start of the second interview the respondents were filtered with the same filter questions as before into one visitor- and one non-visitor-group, regardless of their status in the first interview. Details about the data collection are presented elsewhere [53].

### 2.3. Dropouts

Table 1 shows the number of interviews made at t_1_ and t_2_ as well as the number of dropouts (those who could not be interviewed twice, regardless of reason) at each point in time. Nine calls were made to reach the respondents at t_2_. A comparison with field study samples from the same areas [10,11] indicated that the final visitor samples were representative of the users of the respective area in terms of gender, age, and educational level. To test whether the dropouts differed from the remaining samples of visitors from t_1_ regarding the central variable annoyance with sound from aircraft, *t-*tests were conducted. The *t*-test assesses whether the means of two groups are statistically different from each other. No systematic differences were found. Multivariate logistic regression analyses were conducted to test whether the dropouts from the initial non-visitor group differed systematically from the remaining sample regarding reasons for non-use and earlier experience with the area (see the section “Analyses and Variables” for a description of the variables). Respondents who indicated that a reason for not visiting Bygdøy at t_1_ was that they were not interested in outdoor recreational activities, or they had just moved in, or their health was too bad, were less likely to participate in the second survey than other non-visitors. Non-visitors who preferred other outdoor recreational areas at t_1_, were less likely to drop out of the Bygdøy sample than other non-visitors. Lack of interest in outdoor recreational activities and bad health as reasons for being non-visitors at t_1_ were also associated with a heightened probability to drop out of the Romeriksåsen sample. In addition, the non-visitors at t_1,_ who had visited the area earlier, were more likely to be in the final sample than subjects with no earlier experience with the area.

### 2.4. Analyses and Variables

The analyses were conducted using the statistical package SPSS 9.0 for Windows. The analyses were restricted to the respondents who were interviewed both at t_1_ and t_2_. The samples from the two study areas were analyzed separately, to examine behavioural responses to either a decrease or an increase in noise exposure. The questionnaires for the first and the second interviews were, with one exception, identical until the conclusion of the second interview, which included some additional questions.

#### 2.4.1. Analyses of Behavioural Responses to Decreased Noise Exposure

A paired samples *t*-test was used to compare use frequency at t_1_ and t_2_ among the respondents who visited the area both survey years (*n* = 591). The paired samples *t*-test procedure assesses whether the means of repeated measures for the same units are statistically different from each other. Although the frequency variable was an ordinal and not a true continuous variable, which is formally required to conduct the test, the *t*-test was chosen based on the experience that the *t*-test can be conducted with ordinal variables when there are at least four categories, and the variable is approximately normally distributed. The visitors were asked how often they had engaged in each of a series of activities in the area the past three months. The response categories were: “1–2 times during the past three months”, “1–3 times a month”, “1–2 times a week”, or “more than twice a week”. A single measure of use frequency was obtained by using the respondents’ highest frequency of participation in any of the special activities. For example, if a respondent had used the area for bicycle riding “1–2 times during the past three months” and for hiking “1–2 times a week”, his score on the frequency variable would be “1–2 times a week”. In the analysis the variable was coded from 1 (1–2 times a week) to 4 (more than two times a week).

Multivariate logistic regression analysis was conducted to examine the relationship between reporting aircraft noise to be a reason for non-use at t_1_ and becoming a visitor at t_2_ (*n* = 364, non-visitors at t_1_). The dependent variable was a dichotomous variable indicating whether the respondent was a visitor (1) or non-visitor (0) at t_2_.

Other reasons for non-use at t_1_ were included as independent variables in the analysis to control for other factors possibly constraining use. Other area related reasons than noise were included, both in order to mask the focus on this special issue in the questionnaire, and to be able to control for the influence of other area related issues in the analysis. Likely reasons for non-use, beside noise, were related to the recreational quality of the area, and factors commonly found in the constraints literature [57]. The respondents were asked to indicate whether each statement applied or did not apply to them, or if they were not sure. Very few respondents (on average 2.5%) answered “not sure” to any of these questions, and “not sure” was collapsed with the “does not apply” category for the analysis. The variables were scored 0 (does not apply/not sure), or 1 (applies). Since one of the analyses including these variables was conducted on a relatively small sample (*n* = 69, see below), all reasons could not be included in the analysis as separate variables. A rule of thumb is that no more than *n*/10 variables should be included in a multivariate regression analysis, where *n* is the sample size [58]. “Too much aircraft noise” was included in the analyses as a separate variable, because this was the main variable of interest. “Prefer other areas” was also included as a separate variable since it was assumed that the aircraft noise exposure in the area at t_1_ might be one possible reason for this preference, although of course there might exist many other reasons. The other statements were combined into indices since the variables were not the focus of interest in the context of these analyses, but were included only to control for other possible reasons for non-use. The other area related items were combined to form an additive index labelled “area quality”: “crowding”, “too much careless bicycle riding”, “too much human encroachment”, “vehicles on the walks interfere with the experience”, and “traffic noise from nearby road interferes with the experience”. This additive index has scores from zero to five. The rest of the items were combined thematically into two indices on an intuitive basis. The index “personal reasons” includes the statements “have just moved here”, “poor health”, and “lack of time or opportunity”. The last index was labelled “activity/area relevance”. It includes the items “lack of interest in outdoor recreation”, “do not use the area at this time of the year”, and “it takes too much preparation or travel to go there”. These reasons have to do with the relevance to the respondent of the study area, or the relevance of the special activities that can be conducted in the area, either in general or at the time of the year of the study. The last two indices were scored one if one of the reasons applied, and zero if none applied.

Finally, the variable “earlier experience with the area” was included in the analysis. At t_1_ the non-visitors were asked if they had made use of the area earlier (without any specific time reference). The categories were “yes”, “no” or “don’t know”. Due to very few cases in the last category, the categories “no” and “don’t know” were combined in the analyses and scored 0, while yes scored 1.

To examine the relationship between aircraft noise as a reason for non-use at t_1_ and frequency of use among new visitors at t_2_, a multivariate linear regression analysis was conducted (*n* = 69, non-visitors t_1_, visitors t_2_). Dependent variable was the frequency variable described above. Due to few cases in the two highest categories, these two categories of the frequency variable were collapsed, which means that the variable was scored from 1–3 in this analysis. The independent variables, reasons for non-use and earlier experience with the area, were the same as in the previous analysis.

#### 2.4.2. Analyses of Behavioural Responses to Increased Noise Exposure

A paired samples *t*-test was conducted to compare use frequency at t_1_ and t_2_ among the respondents who visited the area both survey years (*n* = 641). The variable most commonly used to describe experiential effects of noise exposure is “annoyance”. To identify visitors that would be especially sensitive to an increase in noise exposure, an analysis of factors influencing noise annoyance in the recreational area at t_1_ was first conducted. The influence of contextual variables on reactions to noise in recreational settings has been indicated in previous studies of experiential effects [4,6,10,11,59]. To examine the relationship between annoyance with aircraft noise and visitor characteristics, a multivariate linear regression analysis was conducted (*n* = 971, visitors t_1_). Since the use of telephone interviews restricts the number of categories that can be presented per question, the dependent variable, “annoyance with sound from aircraft” was measured on a four-point scale instead of the more commonly used five category annoyance scale [6–8]. The categories were scored from 1–4, from the lowest to the highest degree of annoyance: “not annoyed”, “slightly annoyed”, “rather annoyed” or “very annoyed”. The independent variables described activities, reasons for participation, which part of the area was visited, and frequency of use.

The following activity variables are dichotomous variables describing participation in outdoor activities in Romeriksåsen during the past three months: “bicycle riding”, “picking berries or mushrooms”, “hiking”, “bathing, sunbathing”, “running, jogging”, and “picnic”. The variables were scored one for participated and zero for not participated. In addition, activities with low participation rates were combined and included as indices in the analysis. Participation in one of the activities in the index gives the score one, participation in none gives zero. These indices are “hunting or fishing”, and “other activities” consisting of “orienteering”, “spending the night in a tent or in the open”, “rowing or paddling”, “spending the night in a hut”, and “riding a horse”.

The importance of various reasons for engaging in outdoor recreational activities was measured by two questions. First, the respondents were asked to rate the importance of a series of possible reasons for engaging in outdoor recreational activities. Some of the reasons were adopted from the study by Vaagbø [2]. The reasons listed were: “To experience the silence and peace of nature”, “keeping myself fit”, “to experience birds and animals, flowers and trees”, “to be together with family and friends”, “to get away from hustle and bustle”, “to experience the excitement of hunting or fishing”, “to test my ability to master difficult situations”, “to get away from noise and pollution”, “to experience the greatness of the creation ”, and “other reasons”. The response categories were “very important” (3), “rather important” (2), or “not very important” (1). The non-specific “other reasons” was not included in the analyses, since this is a variable that would be hard to interpret. Second, the respondents were asked about the most important reason for participation. Since the respondents were interviewed on the telephone, they could not be given more alternatives to choose from than they could keep in memory. Therefore, they were asked to choose one out of five broader categories of reasons that were thought to capture the essence of the single reasons in the previous question. The alternatives were: “the nature experience”, “to be together with others”, “the experience of the peace and quiet of nature”, “to keep oneself fit” or, “other reasons”. Each of the alternatives was coded one if it was the most important reason and zero if not. The alternative “other reasons” was not included in the analyses, of the same reason as mentioned above.

The variables describing reasons for participation were combined into additive indices to reduce the number of variables and eliminate possible colinearity problems. Before combining variables, factor analysis was conducted to explore what variables could meaningfully be combined. The unrotated solution was used as basis for the additive indices, since neither orthogonal nor oblique rotation was offering more meaningfully coherent dimensions. Scores on variables with a positive loading higher than 0.50 on a given factor were summed. Addition of variables was chosen instead of the variables directly generated by the factor analysis because additive indices offer scores that are simpler to interpret than the factor scores. Before constructing the indices, the variables were z-transformed to obtain the same standardized scale of scores for all indices.

The index “the nature experience” includes “to experience the silence and peace of nature”, “to experience birds and animals, flowers and trees”, “to get away from hustle and bustle”, “to get away from noise and pollution”, “to experience the greatness of the creation”, and “most important is to experience nature, or the peace and quiet of nature”. The index “fitness” includes “keeping myself fit” and “most important is physical activity”. “Be together with others” includes the variables “to be together with family and friends”, and “most important is to be together with others”. Finally, the index “excitement and mastering” includes “to experience the excitement of hunting or fishing”, and “to test my ability to master difficult situations”. The correlation between the indices was low (≤0.16, Spearman’s rho), and was not thought to cause colinearity problems.

Whereas the change in exposure to aircraft noise was relatively equal in all parts of Bygdøy, it varied between different parts of Romeriksåsen with the largest change in the northern part of the area. It was also assumed that the different parts of Romeriksåsen to some extent were used for different purposes. Therefore, the respondents were asked about the name of the place in Romeriksåsen that they had visited most often during the past three months. For the purpose of our analyses places visited were divided in four categories: “the northern part”, “the middle part”, and “the southern part”, and having visited “more than one part”. Three dummy variables were generated: “Visited only the middle part” (1) *versus* “other parts” (0), “visited several parts” (1) *versus* “other parts” (0), or “visited only the northern part” (1) *versus* “other parts”. Finally, the variable describing frequency of use was included in the analysis.

To examine whether there was a relationship between visitor characteristics at t_1_ and the likelihood of becoming a non-visitor at t_2_ a multivariate logistic regression analysis was conducted (*n* = 971, visitors t_1_). The dependent variable was indicating status in the sample as either non-visitor (1) or visitor (0) at t_2_. Independent variables in the analysis were the same as in the previous analysis that identified characteristics of the visitors who tended to be annoyed by aircraft noise at t_1_.

Finally, to examine whether there was a relationship between aircraft noise as a reported reason for non-use at t_2_ and visitors’ characteristics at t_1_, a multivariate logistic regression analysis was conducted (*n* = 330, visitors t_1_, non-visitors t_2_). The dependent variable in the analysis was aircraft noise as reason for non-use at t_2_, coded one if it applied, and zero for does not apply/not sure. The same independent variables were used as in the two previous analyses.

In the tables throughout the paper that present results from multivariate logistic regression analysis, the odds ratios describe the effect of one unit change in the independent variables. All multivariate regression analyses were conducted in one step. Because the dependent variables used in the multivariate linear regression analyses were ordinal, and not true continuous variables, the suitability of linear models was initially tested by comparing the results to the results of multivariate monotonic regression models. The program GOLDMineR 2.0 from SPSS was used in these analyses. Since the results did not offer any significant improvement over the linear models, the simpler linear models were chosen.

## 3. Results

### 3.1. Effects of Decreased Noise Exposure

#### 3.1.1. Changes in Frequency of Use, Visitors at Both t_1_ and t_2_

A paired samples *t*-test showed no significant change in mean frequency of use among the 591 respondents who had used Bygdøy both before and after the closure of the airport (*t* = 0.62, degrees of freedom = 590, *p* = 0.54). Although there were some changes, there was no systematic change in frequency of use in one particular direction. About one half had used the area at the same frequency as before, one fourth had used it less frequently, and one fourth had used it more often than before.

#### 3.1.2. Aircraft Noise as a Reported Reason for Non-Use at t_1_ and Transition to Use at t_2_

In total, 12.6 percent of the non-visitors (*n* = 364) reported aircraft noise to be a reason for not visiting Bygdøy at t_1_. At t_2_, 0.7 percent of the non-visitors (*n* = 604) stated that this was a reason for not visiting the area. Table 2 shows the results of the logistic regression analysis of the relationship between reporting aircraft noise to be a reason for not visiting Bygdøy at t_1_, and becoming a user of the area at t_2_. The multivariate model was controlled for other reasons for non-use and earlier experience with the area (*n* = 364). The analysis showed no significant relationship between any of the reasons for not visiting Bygdøy at t_1_, and becoming a visitor at t_2_. The only variable with a significant effect was earlier use of the area. The non-visitors at t_1_, who had visited the area earlier, had an increased likelihood of being visitors at t_2_, compared with those who never had visited the area.

#### 3.1.3. Aircraft Noise as a Reason for Non-Use at t_1_, and Frequency of Use among New Visitors at t_2_

Table 3 shows the results of the multivariate linear regression analysis of the relationship between aircraft noise as a reason for non-use at t_1_ and frequency of use at t_2_. Too much aircraft noise as a reason for non-use at t_1_ was the only significant predictor of frequency of use among new visitors at t_2_ (*p* < 0.05). Visitors for whom this was a reason for non-use at t_1_ tended to use the area more frequently at t_2_ than other new visitors.

### 3.2. Effects of Increased Noise Exposure

#### 3.2.1. Changes in Frequency of Use, Visitors at Both t_1_ and t_2_

The frequency of visits to Romeriksåsen among the 641 respondents who had visited the area both years had decreased from t_1_ to t_2_, but not significantly (*t* = 1.84, degrees of freedom = 640, *p* = 0.07). Twenty-nine percent had used the area less and 23 percent more frequently at t_2_. Forty-eight percent had visited the area at the same frequency as at t_1_.

#### 3.2.2. Visitors’ Characteristics and Annoyance with Sound from Aircraft at t_1_

The results of the multivariate linear regression analysis of the relationship between activities, reasons for participation, and annoyance with sound from aircraft at t_1_ are shown in Table 4. The model also included frequency of use, and which part of the area was visited (*n* = 971).

The nature experience as a reason for participation in outdoor recreational activities was indicated to be the strongest predictor of annoyance with sound from aircraft in the model. There was a significant positive relationship between two activity variables, hunting or fishing, and bicycle riding, and annoyance with sound from aircraft. There was also a difference in degree of annoyance depending on what part of the area was visited. The visitors to the northern part of Romeriksåsen were significantly less annoyed by sound from aircraft than other visitors. The northern part was also the part of the area that was least exposed before the change. There was a significant tendency that visitors who had used several parts of Romeriksåsen were less annoyed than visitors who had used only one part of the area.

#### 3.2.3. Visitors’ Characteristics at t_1_, and the Probability of Becoming a Non-Visitor at t_2_

Multivariate logistic regression analysis was used to investigate whether the situational model above could predict a transition from use at t_1_ to non-use at t_2_. The results are shown in Table 5 (*n* = 971). The variables that predicted annoyance with sound from aircraft at t_1_ did not predict a transition from use at t_1_ to non-use at t_2_. None of the variables in the model significantly increased the probability of becoming a non-visitor at t_2_. But there were some groups who had a decreased probability of becoming non-visitors, compared to the others. The likelihood of becoming a non-visitor at t_2_ decreased with increasing use frequency at t_1_. Having participated in the activities of picking berries or mushrooms, or running, jogging, significantly decreased the probability of becoming a non-visitor.

#### 3.2.4. Visitors’ Characteristics at t_1_ and Aircraft Noise as a Reason for Non-Use at t_2_

At t_1_, 5.3 percent of the non-visitor sample (*n* = 399) stated that too much aircraft noise was a reason for not visiting Romeriksåsen. At t_2_, 11.8 percent of the non-visitors (*n* = 643) reported this to be a reason for not visiting. Table 6 shows the relationship between the situational variables measured at t_1_ and too much aircraft noise as a reason for non-use at t_2_ in the restricted sample of new non-visitors at t_2_ (*n* = 330).

Too much aircraft noise was a more likely reason for not using the area at t_2_, if the nature experience itself was an important reason for participation in outdoor recreational activities. Aircraft noise was a more likely reason for non-use at t_2_ for those who had visited the northern part of the area at t_1_ than for other visitors. The respondents who participated in the activity bicycle riding at t_1_ were significantly less likely to blame aircraft noise for not having used the area at t_2_. Finally, too much aircraft noise was a significantly more likely reason for non-use at t_2_ for subjects who had visited the area frequently than for the more infrequent visitors at t_1_.

## 4. Discussion

### 4.1. Summary

No significant differences were found in frequency of use among the respondents who visited either area both before and after the relocation of the main airport. There was no significant indication of a systematic relationship between the altered noise conditions and the overall transitions from non-use to use at Bygdøy, and from use to non-use in Romeriksåsen. The probability of a transition in both cases was positively influenced by the level of prior experience with the area. Earlier experience with the area was the only variable that significantly predicted behavioural change at Bygdøy, while in Romeriksåsen, in addition to use frequency, participation in the activities of picking berries or mushrooms, and running, jogging, also significantly decreased the probability of becoming a non-visitor at t_2_.

When analyzing only the group of new visitors to Bygdøy, and the group who ceased to use Romeriksåsen, some indications of a relationship between altered noise exposure and altered use were found. The new visitors at t_2_ for whom aircraft noise was a reason for none-use at t_1_ tended to use Bygdøy more frequently after the moving of the airport than other new visitors. The visitors to Romeriksåsen most likely to cease using the area because of the increased aircraft noise exposure were visitors to the northern part of the area where the change in exposure was largest, the frequent visitors at t_1_, and visitors for whom the nature experience was an important reason for participation in outdoor recreational activities. The last mentioned variable was the variable that most strongly predicted annoyance with sound from aircraft at t_1_. No firm conclusions can be drawn when it comes to the question of different activities and effects of aircraft noise, since the results are not consistent across the analyses.

### 4.2. The Use of Panel Data

The time perspective of the study might, however, have influenced the behavioural effects that were measured. The effects that were measured are short-term effects. Both surveys at t_2_ concerned the first season after the change corresponding to the time of the first interviews. An effect might have been more distinct the next season, because one could argue that people first would have to experience the new situation before it could affect their behavioural choices. Behavioural adjustments may happen gradually, since it may take some time for the recreationists to find new places to go, and to change their habits. On the other hand, in her study of behavioural consequences of management regulations on mountain camping, Vorkinn [48] found that a substantial number of campers seemed to base their behavioural response on expectations of how the regulations would affect them, rather than on actual experience. Another possibility is that people might habituate to the situation instead of changing their behaviour. These are empirical questions that cannot be answered through the present study.

### 4.3. Frequency of Use Following Decreased Noise Exposure in the Group Who Visited Both Years

The result indicated stability in frequency of use just as much as change in the group who visited Bygdøy both survey years. There were just as many who used the area less frequently as more frequently at t_2_ than at t_1_, which indicates that there was not a systematic relationship between use frequency and the changes in noise exposure in this group of visitors. One reason for this might simply be that the noise exposure did not interfere with the recreational experience of these visitors. But it was shown in another paper analyzing the same data [53] that the degree of annoyance with aircraft noise was high before the moving of the airport. Nearly 50 percent reported to have been rather or very annoyed by the sound from aircraft while visiting the area. The effect of the relocation of the airport on aircraft noise annoyance was marked, almost no one reporting to be rather or very annoyed after the change. Also, about half the sample rated Bygdøy as a better recreational area after the change than it was before. These results indicate that there was a perceived improvement in area qualities following the closing of the airport that potentially could influence use frequency. On the other hand, these visitors already used the area in the situation with high aircraft noise exposure in the area, despite being annoyed by the noise. Although aircraft noise was adversely influencing the recreational experience of these visitors, it seems not to have influenced their behaviour in a similar way. One of the most commonly reported constraints to increased leisure participation is lack of time [57]. Possibly, the visitors to Bygdøy at t_1_ were already participating at the level they preferred or could, so that the improvement of the area quality could not mean much to their overall use frequency. Time constraints and accessibility might possibly also have influenced these visitors’ choice to use the area in the first place, despite being annoyed by the aircraft noise at t_1_.

### 4.4. Aircraft Noise as a Reason for Non-Use at t_1_ and the Prediction of Use at t_2_

The results did not indicate a systematic relationship between reporting aircraft noise to be a reason for non-use at t_1_ and transition to use at t_2_. It seems that the noise exposure was overall not a decisive reason for non-use at t_1_ in our sample. That is not to say that it was not one among several factors influencing behavioural choices. But the decision process regarding leisure participation is complex, with different types of constraints influencing the process at various stages [57]. If the aircraft noise exposure in the area was only one among several factors inhibiting or limiting the use of the area at t_1_, a change in noise exposure alone would not be enough to prompt an overall change in behaviour in this heterogeneous group of non-visitors. But earlier experience with the area significantly increased the probability of a transition to use at t_2_. We did not measure behavioural preference in our study, only reasons for non-use. The earlier use of the area might be interpreted as an indication of preference for using the area.

Also, some of the respondents categorized as non-visitors at t_1_ may actually have used the area during the period asked for. To avoid the absolute accidental visitors, the criterion to be categorized as a visitor in our sample was set at more than one visit during the past three months. Robertson and Regula [51] found evidence of short term fluctuations in the use level that was not due to the social or environmental conditions in the area in their study of the impact of sedimentation on water-based recreation. Our result may also indicate fluctuations in use in the group of infrequent visitors, which were unrelated to the area conditions.

### 4.5. Aircraft Noise as a Reason for Non-Use at t_1_ and Frequency of Use at t_2_

Aircraft noise as a reason for non-use at t_1_ was related to frequency of use at t_2_ when including only those who had become visitors at t_2_. The result of this analysis indicates positive behavioural change following environmental change, which is a kind of change that has barely been studied, as pointed out by Vorkinn [48]. The validity of the result depends on the reasons for non-use being true expressions of the respondents’ motivations. The reasons were formulated by the researchers, not by the respondents. However, they covered a wide range of possibilities, both area related reasons other than aircraft noise, and common constraints to leisure in general [57]. The respondents were given the opportunity to answer “not sure”, but very few were not able to say whether the suggested reasons applied or did not apply to them. Only six out of the 700 non-visitors that were initially interviewed found none of the listed reasons to apply to them. This indicates that the listed reasons covered the respondents’ reasons pretty well. There was no special focus on noise in the introduction of the interview, or in the items presented. Thus, there should be no special reason to state aircraft noise to be a reason for non-use if it was not at all important. The non-significant relationships between use frequency and all other variables in the model were negative, suggesting that these variables limited use frequency for these respondents. While the results indicated behavioural change caused by the altered noise conditions, this supports the interpretation that at least some of the overall transition in use may be explained as short-term fluctuations in use in the group of infrequent visitors, whose use level may be limited by various factors other than noise.

### 4.6. Frequency of Use Following Increased Noise Exposure in the Group Who Visited Both Years

Although the result is parallel to the result regarding decreased noise exposure, the situations facing the visitors are not the same, since the changes in aircraft noise exposure were the opposite. Other factors may explain stability in use in the face of area deterioration, than stability in use following improvement in area quality.

First, although there was no significant change in use frequency among those who used Romeriksåsen both before and after the increase in aircraft noise exposure, some kinds of behavioural adaptation in this group of visitors cannot be totally ruled out. A more sophisticated model could possibly have detected other types of behavioural adaptation. Our analysis could not detect temporal displacement, or intra-site displacement. Both temporal and intra-site displacement might have been feasible strategies in Romeriksåsen, and consistent with our findings of no systematic change in use frequency. Previous studies on coping with perceived adverse area factors have found that the use of behavioural adaptation within recreational areas may be pervasive [37,38,42,46]. For instance, Anderson and Brown [37] found that over time, a majority of the respondents either changed their entry points to the Boundary Waters Canoe Area Wilderness, or selected campsites differently, or entered on a different day of the week. Hall and Shelby [38] found that about half the visitors in their survey altered behaviour in response to crowding at the Lake Billy Chinook reservoir. They found temporal displacement to be the most common strategy, but intra-site displacement was also common. In a explorative study of coping in the Adirondack wilderness area Johnson and Dawson [42] also found that about half their respondents used some kind of coping strategies. In a cross sectional survey including local residents of the communities in and around the Acadia National Park [46] most community residents reported that they continued to use the carriage roads of the park at about the same level as before, despite increased use levels and problem behaviour. However, the authors [46] also found that almost all respondents used some kinds of coping strategies. The two last mentioned studies [42,46] examined the use of both behavioural and cognitive coping mechanisms. Temporal and intra-site displacement were the coping strategies reported most frequently in both studies. Both studies also found that a majority used more than one strategy in response to crowding or conflict [42,46]. Johnson and Dawson state that: “coping is more than just a singular decision, but a complex process that is part of a larger, flexible user decision-making strategy.” [42], p. 288.

Cognitive coping strategies were not examined in our study, but is another set of coping strategies that would be consistent with continued use in the face of perceived adverse changes in area conditions (e.g., [42,45–47,49,50]). The following description of the cognitive coping strategies of product shift and rationalization is given by Miller and McCool ([45], p. 262), with reference to Shelby *et al.* ([50], p. 276): “Product shift involves a “change in the definition of the experience and Standards for the importance of characteristics of that experience.” Product shift also represents an overall change in the definition of the area. The net result of this is that satisfaction remains high and recreationists are not obliged to remove themselves either physically or temporally from the area. Rationalization represents a process whereby recreationists re-evaluate an undesirable situation in a more favourable light.” Although the existence of cognitive adjustments cannot be ruled out in our study, there are some indications of the opposite. It was shown in another paper analyzing the same panel data that the respondents who visited both at t_1_ and t_2_ perceived the quality of the recreational area to have deteriorated after the change due to the increased aircraft noise exposure in the area [53]. That is, it seems that this group of visitors tends to continue to use the area as they used to, despite the perceived deterioration of the area quality caused by the increased aircraft noise exposure.

The finding is similar to results in Vorkinn’s panel study [48] of visitor response to management regulations of “wild camping”. She found that a relatively high percentage of the existing users who revisited the site after the regulations were dissatisfied. Despite that, the experienced users were more likely to adapt to the new situation, than to give up camping in the area. Vorkinn ([48], p. 744), suggests that “a possible explanation for this is that people develop emotional bonds through repeated use of an area, which gives the area/site a value or meaning that other areas/sites do not have. If this is the case, alternative areas with similar environmental attributes will *not* be experienced as real substitutes.” The existence and consequences of emotional bonds to, and dependence on, specific sites or areas have been examined under the label “place attachment” [48,60–64]. A relationship between place attachment and continued use in the face of real or potential adverse changes in area conditions has also been indicated by others [40,65,66]. More specifically, there are indications that place attachment may be an especially important factor in regard to behavioural responses to adverse changes in the quality of local recreational areas [66]. Attachment to outdoor recreational areas has among other things been found to be related to the proximity of the area to the homes of the visitors and use frequency [48,61–64,66]. Frequency of use at t_1_ was adversely associated with the likelihood of becoming a non-visitor at t_2_, which indicates that the people who visited both years tended to be the most frequent visitors in our sample. While place attachment was not directly measured in our study, the results indicate that this variable should be included in future studies of noise impacts in local recreational areas. Constraints related to distance from home and travel time may further limit the substitutability of local areas, despite perceived adverse area conditions [39,40].

### 4.7. Characteristics of Visitors Predicting Aircraft Noise Annoyance at t_1_

The purpose of this analysis was to identify the visitors at t_1_ most vulnerable to aircraft noise, to examine the possible relationship between vulnerability to aircraft noise and behavioural change at t_2_, not to examine what variables predict noise annoyance in an outdoor recreational situation per se. The geographical variables are specific to this particular area. The noise situation was a particular low noise exposure situation. Also, both annoyance and visitors’ characteristics were measured in a rather broad sense, encompassing the experiences and behaviours of a three months period. The *R^2^* was low, which indicates that the variables in the model explained only a small amount of the variance in noise annoyance. One variable obviously lacking was individual noise exposure, which was not obtainable for this study. The result that annoyance with aircraft noise in the recreational area was positively related to the nature experience as a reason for participation in outdoor recreational activities is, however, in accordance with previous findings [10,11]. The result that the visitors to the northern part of the area were significantly less annoyed than other visitors is in accordance with what one would expect from the exposure situation at t_1_, which validates the result.

### 4.8. Visitor Characteristics at t_1_ and the Probability of Becoming a Non-Visitor at t_2_

The variables that predicted annoyance with aircraft noise at t_1_ did not predict transition from use to non-use at t_2_. Instead, frequency of use and participation in the activities “picking berries or mushrooms”, and “running, jogging” at t_1_ were associated with a decreased probability of ceased use at t_2_. Our result regarding frequency of use is in line with findings in Vorkinn’s study [48] of regulations on wild camping in a Norwegian mountain area. The more experience the respondents had in the area, the more likely they were to adapt to the new situation and stay at a mountain camp, instead of ceasing to camp in the area. Vorkinn suggests that place attachment may be a possible explanation for this finding, the emotional bonds giving the area a special value or meaning that makes it hard to find a substitute area. The possible role of place attachment in our study was discussed above. Frequency of use may indicate place attachment, and place attachment is shown to be related to a decreased probability of ceased use. Hall and Shelby [38] found that a higher percentage of the most experienced users of a large reservoir, measured in year since first visit to the area, used temporal displacement, or a combination of temporal and spatial displacement than the less experienced visitors. Spatial strategies alone where most often used by the least experienced visitors. Although “frequency of use” the past three months and “time since first visit” are measuring different aspects of experience, the results from our study and the study of Hall and Shelby [38] are in line, in that the more experienced visitors seem to be less likely to use the most extreme coping response, being displaced from the site altogether. “Picking berries or mushrooms” is a kind of activity that obviously is dependent on special area resources, which is a likely reason for the decreased probability of ceased use for those who participated in this activity. “Running, jogging” is an activity that one would assume to be less sensitive to the experiential qualities of the area, and more focused on practical aspects like accessibility and suitability for the activity.

### 4.9. Visitor Characteristics at t_1_ and Aircraft Noise as a Reason for Non-Use at t_2_

The two variables that were shown to significantly increase the likelihood of reporting aircraft noise to be a reason for non-use at t_2_ were also significantly related to aircraft noise annoyance at t_1_. Visitors to the northern part of the area tended to be less annoyed by aircraft noise at t_1_, but more likely than other visitors to report aircraft noise to be a reason for ceased use at t_2_. The result is plausible, since the actual change in aircraft noise exposure was estimated to be largest in the northern part of Romeriksåsen. Apart from the larger increase in aircraft noise exposure in the northern part than in the other parts, interference from aircraft noise might also have been perceived as stronger in the northern part because this part of the area was the most pristine in other ways. The nature experience as a reason for participation in outdoor recreational activities was the variable indicated to have the largest effect on noise annoyance at t_1_, and was also related to increased probability of reporting aircraft noise as a reason for non-use at t_2_. Ceased use at t_2_ was, overall, shown to be less likely for the more frequent visitors at t_2_ than for the more infrequent visitors. But some of the frequent visitors did also become non-visitors. Too much aircraft noise was a more likely reason for ceased use at t_2_ for the previously frequent visitors than for those who visited more infrequently at t_1_. One of the previously frequent visitors expressed “deep deep sorrow because of all the aircraft noise that has destroyed my favourite outdoor recreational area.” The statement was given in response to an open-ended question where the non-visitors were asked to elaborate on “other reasons” for non-use at t_2_. This particular person visited more than twice a week at t_1_, but had not visited at all the past three months at t_2_. The statement indicates place attachment and serious impact for this particular previous visitor. Although only the statement of one visitor, it supports the suggestion that place attachment may be an important variable to include in future studies of noise impacts in local outdoor recreational areas. Overall, the results show that although there was no systematic cessation in use due to aircraft noise, to some the character of the area was changed in a way that appears to hamper their goal attainment in the area.

## 5. Concluding Remarks

The present paper examined the impact of environmental change at the individual level, utilizing panel methodology. The results demonstrated that changed aircraft noise exposure may influence individual choices to use local outdoor recreational areas. However, considerable stability in use, and also fluctuations in use unrelated to the changes in noise conditions were demonstrated. Worth noting is the combination of considerable experiential impact, found in another paper analyzing the same data, and the relative modest behavioural response to the change found in this paper. This combination of impacts suggests that careful considerations are needed in the planning of air routes over local outdoor recreational areas. It would be especially helpful if future studies were designed to examine a broader set of coping mechanisms, like intra-site and temporal displacement. Future studies of behavioural responses to noise in local recreational areas should also consider the effects of place attachment, and the perceived substitutability of the area.

## Figures and Tables

**Table 1:** Interviews and dropouts at t_1_ and t_2_, Bygdøy and Romeriksåsen.

	Bygdøy				Romeriksåsen			
	Visitors t_1_		Non-visitors t_1_		Visitors t_1_		Non-visitors t_1_	
		
	1,600		700		1,620		700	
	Vis. t_2_^1^	Non-vis. t_2_	Vis. t_2_	Non-vis. t_2_	Vis. t_2_	Non-vis. t_2_	Vis. t_2_	Non-vis. t_2_
	591	309	69	295	641	330	86	313
Sum t_2_	900		364		971		399	
Dropouts:								
Refused t_1_^2^	216		145		85		73	
Lost t_2_^3^	434		157		483		167	
Refused t_2_^4^	50		34		81		61	

1 Vis. = Visitors; Non-vis. = Non-visitors.

2 Refused t_1_ = Refused contact information to be recorded at the end of the first interview.

3 Lost t_2_ = People who either did not answer the phone at t_2_, or the contact information was wrong.

4 People who refused to be interviewed a 2nd time when contacted at t_2_.

**Table 2:** Probability of becoming a visitor at t_2_ dependent on reasons for not visiting Bygdøy at t_1_, and earlier experience with the area^1^.

Variables in the model	OR 2	95% CI 3 for OR
Too much aircraft noise	1.584	0.704–3.565
Prefer other areas	0.731	0.408–1.312
Personal reasons	1.257	0.690–2.290
Activity/area relevance	0.980	0.540–1.776
Area quality	1.035	0.789–1.357
Earlier experience with the area	3.620**	1.491–8.792

1 Multivariate logistic regression model (*n* = 364, ** −*p* < 0.01).

Dependent variable is visitor (1) or non-visitor (0) at t_2_.

Hosmer and Lemeshow test of goodness-of-fit: Chi-Square = 0.592, degrees of freedom = 7, *p* = 0.710.

2 OR = Odds Ratio.

3 CI = Confidence Interval.

**Table 3:** Frequency of use of Bygdøy at t_2_ dependent on reasons for not visiting at t_1_, and earlier experience with the area^1^.

Model (adj. *R2* = 0.126)	Unst.B 2	SE 3	Beta 4	*t*^5^
Constant	0.988	0.350		2.83
Too much aircraft noise	0.704	0.219	0.400*	3.22
Prefer other areas	−0.133	0.159	−0.101	−0.83
Personal reasons	−0.006	0.172	−0.004	−0.03
Activity/area relevance	−0.040	0.167	−0.028	−0.24
Area quality	−0.122	0.079	−0.190	−1.54
Earlier experience with the area	0.457	0.262	−0.200	1.74

1 Multivariate linear regression model (*n* = 69, *−*p* < 0.05).

Dependent variable is frequency of use of Bygdøy at t_2_.

2 Unstandardized Beta.

3 Standard Error of the Unstandardized Beta.

4 Standardized Beta.

5 The *t*-score of the linear regression *t*-test.

**Table 4:** Annoyance with sound from aircraft at t_1_, Romeriksåsen, dependent on visitors’ characteristics^1^.

Model (adj. *R2* = 0.039)		Romeriksåsen t_1_ (*n* = 971)			
		Unst. B 2	SE 3	Beta 4	*t*
Constant		1.289	0.114		11.36

Activities	Hunting or fishing	0.201	0.074	0.109**	2.71
	Bicycle riding	0.126	0.061	0.068*	2.05
	Picking berries or mushrooms	0.079	0.060	0.044	1.32
	Hiking	0.115	0.087	0.044	1.32
	Bathing, sunbathing	0.075	0.061	0.042	1.23
	Running, jogging	0.022	0.073	0.010	0.31
	Picnic	−0.007	0.061	−0.004	−0.11
	Other activities	0.010	0.068	0.005	0.14

Reasons for Participation	The nature experience	0.027	0.008	0.117***	3.54
	Excitement and mastering	−0.002	0.021	−0.003	−0.08
	Fitness	−0.021	0.018	−0.039	−1.13
	Be together with others	−0.025	0.018	−0.045	−1.38

Other variables	Frequency of use	0.013	0.032	0.014	0.41
	Visited only the middle part	−0.079	0.068	−0.043	−1.16
	Visited several parts	−0.168	0.084	−0.070*	−2.00
	Visited only the northern part	−0.268	0.093	−0.101**	−2.88

1 Multivariate linear regression model (*n* = 971, *−*p* < 0.05; **−*p* < 0.01; ***−*p* < 0.001).

Dependent variable is annoyance with sound from aircraft at t_1_.

2 Unstandardized Beta.

3 Standard Error of the Unstandardized Beta.

4 Standardized Beta.

**Table 5:** Non-use at t_2_, dependent on activities, reasons for participation, frequency of use, and what part of Romeriksåsen was visited at t_1_^1^.

	Variables in the model	OR 2	95% CI 3 for OR
Activities	Hunting or fishing	1.007	0.687–1.475
	Bicycle riding	0.786	0.569–1.086
	Picking berries or mushrooms	0.619**	0.459–0.835
	Hiking	1.477	0.940–2.321
	Bathing, sunbathing	1.101	0.802–1.512
	Running, jogging	0.664*	0.445–0.989
	Picnic	0.805	0.588–1.102
	Other activities	0.933	0.653–1.335

Reasons for participation	The nature experience	0.973	0.936–1.012
	Excitement and mastering	1.019	0.914–1.136
	Fitness	1.009	0.920–1.108
	Be together with others	1.042	0.951–1.143

Other variables	Frequency of use	0.657***	0.556–0.777
	Visited only the middle part	0.957	0.675–1.355
	Visited several parts	1.028	0.667–1.583
	Visited only the northern part	1.505	0.948–2.389

1 Multivariate logistic regression analysis (*n* = 971, **−*p* < 0.01 ***−*p* < 0.001).

Dependent variable is non-visitor at t_2_ (1) or visitor at t_2_ (0). Hosmer and Lemeshow test of goodness-of-fit: Chi-Square = 6.931, degrees of freedom = 8, *p* = 0.544.

2 OR = Odds Ratio.

3 CI = Confidence Interval.

**Table 6:** Aircraft noise as a reported reason for not visiting Romeriksåsen at t_2_ dependent on visitors’ characteristics at t_1_^1^.

	Variables in the model	OR 2	95% CI 3 for OR
Activities	Hunting or fishing	1.188	0.467–3.025
	Bicycle riding	0.236**	0.080–0.696
	Picking berries or mushrooms	0.964	0.471–1.973
	Hiking	1.076	0.329–3.519
	Bathing, sunbathing	1.210	0.564–2.598
	Running, jogging	1.553	0.542–4.445
	Picnic	0.647	0.283–1.475
	Other activities	1.455	0.593–3.569

Reasons for Participation	The nature experience	1.171**	1.051–1.305
	Excitement and mastering	0.855	0.659–1.109
	Fitness	0.987	0.795–1.226
	Be together with others	0.983	0.781–1.238

Other variables	Frequency of use	1.476*	1.001–2.176
	Visited only the middle part	0.689	0.282–1.686
	Visited several parts	0.506	0.151–1.691
	Visited only the northern part	2.522*	1.035–6.149

1 Multivariate logistic regression analysis (*n* = 971, **−*p* < 0.01 ***−*p* < 0.001).

Dependent variable is aircraft noise as a reason for not visiting Romeriksåsen at t_2_.

Hosmer and Lemeshow test of goodness-of-fit: Chi-Square = 7.014, degrees of freedom = 8, *p* = 0.535.

2 OR = Odds Ratio.

3 CI = Confidence Interval.
